# Expansion and herniation: evaluation of the best pregnancy rate predictor after quarter laser assisted hatching in frozen blastocyst transfers

**DOI:** 10.5935/1518-0557.20190090

**Published:** 2020

**Authors:** Caroline Brogliato, Janaína Romanini, Caroline Z Berton, Claudia H Suganuma, Laura T Vellez, Ivan H Yoshida, Caio P Barbosa

**Affiliations:** 1 Instituto Idéia Fértil de Saúde Reprodutiva, Santo André, SP, Brazil

**Keywords:** hatching, blastocyst, thaw, expansion, pregnancy

## Abstract

**Objective:**

To assess the recovery of thawed blastocysts submitted to quarter laser assisted hatching and examine potential correlations between the procedure and pregnancy rates.

**Methods:**

This cross-sectional study included only single-blastocyst transfers performed from July 2017 to December 2018. A total of 765 blastocysts were thawed and immediately submitted to quarter laser assisted hatching in the zona pellucida; they were subsequently incubated for three hours until transfer time, at which time they were examined for collapse or expansion; expanded blastocysts were further evaluated for herniation. The Chi-square test was used in statistical analysis.

**Results:**

627 blastocysts expanded (81.9%) and yielded a pregnancy rate of 40% (251/627). 138 blastocysts collapsed after thawing (18.0%) and yielded a pregnancy rate of 25.4% (35/138) (*p*=0.001). Additional analysis of the subgroup of expanded blastocysts revealed that the 385 herniated blastocysts (61.4%) yielded a pregnancy rate of 43.9% (169/385). The remaining 242 non-herniated blastocysts (38.6%) yielded a pregnancy rate of 33.9% (82/242) (*p*=0.013). Statistical significance was attributed to events with a *p*<0.05.

**Conclusion:**

Quarter laser assisted hatching is a safe, valid, and relatively easy-to-use procedure for thawed blastocysts. Blastocysts that expanded and herniated after quarter laser assisted hatching presented statistically superior results.

## INTRODUCTION

Considered a major breakthrough in assisted human reproduction, embryo vitrification followed by frozen embryo transfer (FET) became widely disseminated and yielded positive effects in the form of increased pregnancy rates (Veleva *et al*., 2013). This technique may be an effective alternative for future embryo transfers, since the procedure, unlike the slow freezing process, avoids the formation of intra- and extracellular ice crystals, which may damage cell membranes and organelles. High concentration cryoprotectants and ultra-fast embryo freezing were developed to avoid possible cell damage (Liang & Montan, 2016; Papadopoulos *et al*., 2002). Despite some variability in results, survival rates after thawing have been acceptable, since the technique is a simple and effective procedure (Dobrinsky, 2002).

Embryo morphology (Ziebe *et al*., 2003), advanced maternal age, and cryopreservation technique have been linked to adverse effects on the hatching of the zona pellucida (Cohen *et al*., 1992) and impaired endometrium receptivity (Ewards *et al*., 1984; Simon & Laufer, 2012), which combined or alone affect embryo implantation.

Opening the zona pellucida is an intentional rupture event designed to facilitate the implantation of the embryo onto the endometrium (Cohen *et al*., 1992). There are different ways to open the zona pellucida, which include mechanical, chemical, and laser-based methods (Antinori *et al*., 1996). Laser has advantages over chemical and mechanical procedures, since it provides objective access to the target via the light beam and precise control to open the zona pellucida without thermal or mutagenic side effects (Germond *et al*., 1995). Lasers, too, are more accessible and adaptable to inverted microscopes (Mantoudis *et al*., 2001).

Assisted laser hatching can be divided into three methods, according to the degree of invasiveness. Perforation may cross the zona pellucida completely, be partial, or not reach the inner membrane. Laser can also be used to thin a large portion of the outside part of the zona pellucida to facilitate hatching, in a procedure known as quarter laser assisted hatching (Davidson *et al*., 2018).

Older patients may benefit from assisted hatching. According to Kilani *et al*. (2006), patients aged 38+ years have denser egg coats. The procedure may also be indicated to individuals with high FSH levels; patients with a history of implantation failure (Cohen *et al*., 1990; 1992); and women undergoing thawed embryo transfers, since cryopreservation induces a microstructural change in the zona pellucida (Moreira da Silva & Metelo, 2010) and adversely affects hatching (Kanyo *et al*., 2016). Balaban *et al*. (2002) and Ng *et al*. (2005) also suggested that opening the zona pellucida of thawed embryos improves embryo implantation since it facilitates the hatching of the zona pellucida.

This study aimed to evaluate the recovery of thawed blastocysts submitted to quarter laser assisted hatching and examine potential correlations with pregnancy rate.

## MATERIALS AND METHODS

This cross-sectional study included only single cryopreserved blastocyst transfers performed from July 2017 to December 2018 at the Instituto Ideia Fértil de Saúde Reprodutiva.

A total of 765 blastocysts were thawed and immediately submitted to quarter laser assisted hatching of the zona pellucida. The blastocysts were cultured for three hours, at the end of which they were assessed for expansion and herniation, and transferred. The blastocysts were categorized as collapsed or expanded; expanded blastocysts were further divided into herniated and non-herniated blastocysts.

Patients given thawed euploid embryos submitted to pre-implantation genetic testing and patients given embryos thawed on the third day of development were excluded.

### Laboratory Reproductive Variables

The following laboratory reproductive variables were considered for thawed embryos:

- Blastocyst expansion;
- Blastocyst herniation (yes/no);
- Morphology grading.

### Statistical analysis

Statistical analysis was performed using the Chi-square test.

## RESULTS

Our study looked into the performance of quarter laser assisted hatching in thawed blastocysts. A total of 765 blastocysts were thawed. More than four in five (627/765) expanded after the procedure and yielded a pregnancy rate of 40% (251/627). The remaining 138 blastocysts collapsed after thawing (18%), and yielded a pregnancy rate of 25.4% (35/138) (*p*=0.001).

In the group of expanded blastocysts, 385 herniated (61.4%) and yielded a pregnancy rate of 43.9% (169/385). The 242 blastocysts that did not herniate (38.6%) yielded a pregnancy rate of 33.9% (82/242) (*p*=0.013).

In terms of age groups, the expanded blastocysts of women aged 35 years or younger did not yield significantly different pregnancy rates whether they herniated (46.05%) or not (41.3%) (*p*=0.381). Expanded herniated blastocysts of patients aged 36 or older outperformed non-herniated blastocysts (41.2% *vs.* 24%) (*p*=0.004).

Surviving blastocysts that remained collapsed after three hours yielded a pregnancy rate of 25.4%, regardless of patient age. However, embryo morphology proved relevant at delivering higher pregnancy rates. Patients whose blastocysts were graded as top quality according to the classification described by Gardner *et al*. (2000) yielded a pregnancy rate of 55.0% (11/20), while lower grade blastocysts yielded a pregnancy rate of 20.3% (24/118) (*p*=0.001).

## DISCUSSION

The zona pellucida is a glycoprotein coat that surrounds the embryo (Zeng *et al*., 2018). It may undergo biochemical changes induced by factors such as advanced maternal age, cryopreservation, and *in vitro* culture, which may make it harder and hamper implantation (Kanyo *et al*., 2016).

Vitrification is considered one of the most significant achievements in assisted human reproduction of the last decade (De Munk & Vajta, 2017). It is a safe method that preserves the quality of embryos after thawing. The stiffening of the zona pellucida after thawing hinders blastocyst hatching, in itself an important process for implantation to occur (Cohen *et al*., 1990). Artificial rupture of the zona pellucida is known as assisted hatching. Most assisted reproduction centers have used the laser-based method because it is a fast, safe, and accurate procedure (Lu *et al*., 2019).

The implantation window is a critical moment when the endometrium reaches optimal receptivity for implantation. Precise synchronization between embryo and endometrium is decisive. The purpose of opening the zona pellucida is to allow the molecules of the endometrium to get in early contact with the embryo, to improve the communication between the two, and increase implantation and pregnancy rates (Mantoudis *et al*., 2001). This study verified that blastocysts that were expanded and herniated after the thawing process attained higher pregnancy rates, corroborating the findings of Edi-Osagie *et al*. (2003). The authors found that assisted hatching presented significant benefit by improving the pregnancy rates of women with a history of implantation failure in particular. They suggested that assisted hatching might increase the chances of pregnancy for these patients.

The study by Elnahas *et al*. (2018) also reported statistically superior results in terms of implantation and pregnancy rates from thawed embryos submitted to assisted hatching. Lu *et al*. (2019) performed a retrospective analysis involving 415 thawed embryo transfers, in which 225 underwent assisted hatching and 190 were in the control group (without assisted hatching). Assisted hatching led to statistically superior results, particularly in terms of clinical pregnancy and implantation rates. The meta-analysis by Zeng *et al.* (2018) included 12 randomized controlled trials (over 2574 participants) and demonstrated that assisted hatching was associated with higher implantation and clinical pregnancy rates, in addition to a higher incidence of multiple pregnancies in women receiving thawed embryos. Mantoudis *et al*. (2001) found higher implantation and pregnancy rates using the quarter laser assisted hatching technique in comparison with embryos in which total assisted hatching was offered to patients meeting at least one of the following criteria: advanced age, prior implantation failure, poor response, or frozen embryos. Corroborating this study, Petersen *et al*. (2005) observed that quarter laser assisted hatching was a good strategy to improve implantation rates in patients with history of implantation failure.

Assisted hatching improved the implantation and pregnancy rates of thawed embryo transfers. However, factors such as embryo morphology grading; hatching potential; endometrial quality; and patient intrinsic traits including anxiety, stress, thrombophilia, endometriosis, adenomyosis, hydrosalpinx and others, are pivotal to treatment success and must be considered during the course of treatment, so that, together with the procedures performed inside and outside the IVF laboratory, we may help our patients achieve pregnancy.

## CONCLUSION

Quarter laser assisted hatching is a safe, valid, and relatively easy-to-use procedure for thawed blastocysts. Blastocysts that expanded and herniated after quarter laser assisted hatching presented statistically superior results.
